# An Acylhydrazone Fluorescent Sensor: Bifunctional Detection of Thorium (IV) and Vanadyl Ions over Uranyl and Lanthanide Ions

**DOI:** 10.3390/ijms26073231

**Published:** 2025-03-31

**Authors:** Xin Lin, Hua Liang, Ke Dai, Jing Zhou, Qiang Tian, Yuge Xiang, Zhicheng Guo, László Almásy

**Affiliations:** 1 State Key Laboratory of Environment-Friendly Energy Materials, School of Materials and Chemistry, Southwest University of Science and Technology, Mianyang 621010, China; 2 School of National Defense, Southwest University of Science and Technology, Mianyang 621010, China; 3 Institute for Energy Security and Environmental Safety, HUN-REN Centre for Energy Research, Konkoly-Thege Miklós út 29-33, 1121 Budapest, Hungary

**Keywords:** fluorescent sensor, thorium recognition, vanadyl response, nuclide ions

## Abstract

Thorium is a notable candidate for resolving uranium shortage caused by the global application of nuclear power generation. Uranium extraction from seawater is another attempt to handle its source deficiency, however, vanadium is one of the main competitive elements in that process. Exploration of probes which can discriminatively detect thorium and vanadium from uranium has primary significance for their further separation and for environmental protection. Herein, N′-(2,4-dihydroxybenzylidene)-4-hydroxylphenylhydrazide, **AOH**, is used as sensor for Th^4+^ and vanadyl (VO^2+^) determination. **AOH** demonstrates a specific “turn-on” fluorescence selectivity towards Th^4+^ over f-block and other foreign metal ions, with a detection limit (LOD) of 7.19 nM in acidic solution and a binding constant of 9.97 × 10^9^ M^−2^. Meanwhile, it shows a “turn-off” fluorescence response towards VO^2+^ over other metal ions at the coexistence of Th^4+^, with a LOD of 0.386 μM in the same media and a binding constant of 4.54 × 10^4^ M^−1^. The recognition mechanism, based on HRMS, ^1^H NMR, and FT-IR results, demonstrates that VO^2+^ causes the fluorescence quenching by replacing Th^4+^ to coordinate with **AOH**. In real water detection tests, Th^4+^ and VO^2+^ exhibited satisfying recoveries. These findings expand the application of sensors in nuclide pollution control.

## 1. Introduction

Thorium is a natural radioactive element and it has a quantity roughly 3 to 4 fold greater than that of uranium in the Earth’s crust, which makes it an alternative nuclear energy source owing to the insufficiency of uranium [1,2,3]. Meanwhile, thorium is known as a strategic element in a variety of industries. However, thorium contributes to radioactive contamination, mainly because of the processing and mining activity of rare earth elements (or lanthanide elements, Ln), owing to the wide range of applications of Ln in many fields, such as magnetic and optical materials [4,5]. Ln are typically found in conjunction with uranium and particularly thorium due to their similar ionic radii [6]. The detection and separation of lanthanide and actinide elements in nuclear waste are one of the most challenging tasks, due to a great similarity in their physical and chemical properties [7]. Th(IV) is the most stable form of thorium in nature and solution. Effective determination of Th^4+^ in coexistence with uranyl and Ln^3+^ ions is important in nuclear waste liquid control and in their further separation and recycling. Instrumental analysis methods such as X-ray fluorescence spectrometry (XRF) [8], gamma spectrometry, inductively coupled plasma mass spectrometry (ICP-MS) [9], and neutron activation analysis (NAA) [10,11,12], have high precision and sensitivity in nuclide ions analysis, yet shortcomings like laborious sample preparation and expensive instruments make them unsuitable for on-site analysis. Fluorescence spectrometry is an exception though it is an instrumental analysis method. It has significant advantages like fast response time, cost-effectiveness, low detection limits with high sensitivity, and selectivity, provided that a suitable fluorescent sensor is chosen [13,14]. Fluorescence sensors have been proven to be excellent luminescent probes toward Th^4+^ [15,16,17,18]. Nevertheless, the issues of anti-interference, water compatibility, and simple sensors preparation are yet to be improved [19,20,21].

In addition to exploring the substitution of uranium, its extraction from seawater is another possibility to solve its shortage, as for the amount of uranium, though with a very low concentration of 3–9 μg/L, it is thousands of times greater than that in the Earth’s crust [22,23]. However, in that process, vanadium is an important competitive element [24,25]. Vanadium exists usually in the form of trace elements and is not essential for most living organisms. In aqueous solution, vanadium mainly exists in +4 and +5 valence states. Its effect on organisms is still controversial yet with potential hazards [26]. Compared with other harmful heavy metals, the detection methods for vanadium are very limited, in spite of its importance in the steel industry, medical treatment, batteries, and other fields [27,28]. A few pioneering works have been done on vanadyl sensing [29,30]; however, its distinguishing detection from uranium are still to be explored [31]. From the studies on uranium pollution control and recovery reported, thorium and vanadium are two interfering elements [25,32], but the determinations of them from uranium at the same time, to our knowledge, have not been considered.

In this work, a simple acylhydrazone, **AOH**, studied earlier as a pharmacophore [33,34], has been investigated for its detection abilities toward Th^4+^ and VO^2+^ ions. Here, **AOH** shows a fluorescence “turn-on” to Th^4+^, and “turn-off” for VO^2+^ with the help of Th^4+^ in acidic aqueous solution even at the co-existence of UO_2_^2+^ and rare earth metal ions. The detection limit and detection mechanism were also investigated. In addition, the detections of Th^4+^ and VO^2+^ in real water were investigated to check the sensor practicability.

## 2. Results and Discussion

### 2.1. Selectivity of AOH Towards Th^4+^

**Figure 1:**
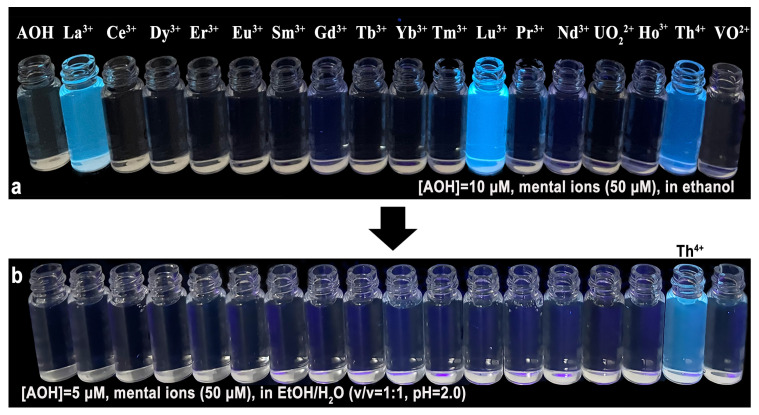
Images of **AOH** before and after the addition of metal ions under 365 nm UV light in (**a**) EtOH and in (**b**) EtOH/H_2_O (1/1, *v*/*v*, pH = 2.0).

The selectivity response of **AOH** to Ln^3+^ and the nuclides Th^4+^ and UO_2_^2+^ were tested preliminarily in pure ethanol. In that medium, it exhibited blue luminescence to La^3+^, Lu^3+^, and Th^4+^ (Figure 1a). When changing the medium from pure ethanol to EtOH/H_2_O (1/1, *v*/*v*, pH = 2.0), the detection selectivity was improved and **AOH** only exhibited strong response to Th^4+^ with no sensitivity to La^3+^ and Lu^3+^ (Figure 1b).

The pH effect investigations show that at pH = 2.0 (50% EtOH and 50% H_2_O in volume, Figure S3), **AOH** had the most sensitive “turn-on” fluorescence response intensity toward Th^4+^. As pH 2.0 is a very acidic condition that may affect the stability of the sensor, the change of the UV-vis spectra of **AOH** with time in EtOH/H_2_O (1/1, *v*/*v*, pH = 2.0) was analyzed to check its stability. (Figure S4). The absorbance of **AOH** at 326 nm decreased slightly and there was nearly no change between 350 nm and 600 nm in one hour (1 h), indicating the good stability of **AOH** in such media at the tested time. Since all the fluorescence testing was performed in less than 1 h with the excitation wavelength at 374 nm, under conditions where **AOH** was stable, the subsequent experiments were conducted under such media unless otherwise noted.

**Figure 2:**
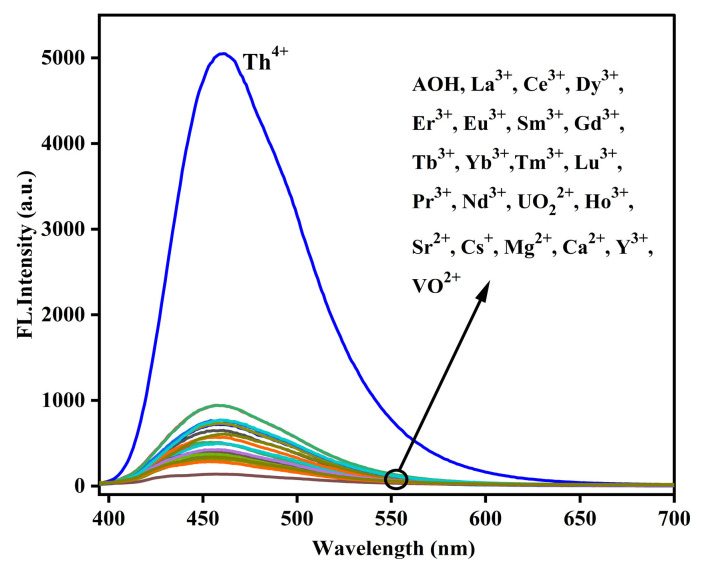
Fluorescence spectra of **AOH** (5 μM) before and after metal ions (50 μM) addition in EtOH/H_2_O (1/1, *v*/*v*, pH = 2.0).

The fluorescence spectra of **AOH** to Th^4+^, UO_2_^2+^, and lanthanide ions (Ln^3+^) with some alkaline earth metal ions show the sensor response uniqueness toward Th^4+^ (Figure 2). That specific selectivity is an improvement over reported Th^4+^ sensors, which are susceptible to the interference from UO_2_^2+^ and some Ln^3+^ [35,36,37]. When common metal ions (Na^+^, Fe^3+^, Ni^2+^, Zn^2+^, Cu^2+^, Al^3+^) were added in the **AOH** solution, no luminescence except that of Th^4+^ was observed (Figure S5), reproving the specific selectivity to Th^4+^.

### 2.2. Interfering Ions Effect on Th^4+^ Response

**Figure 3:**
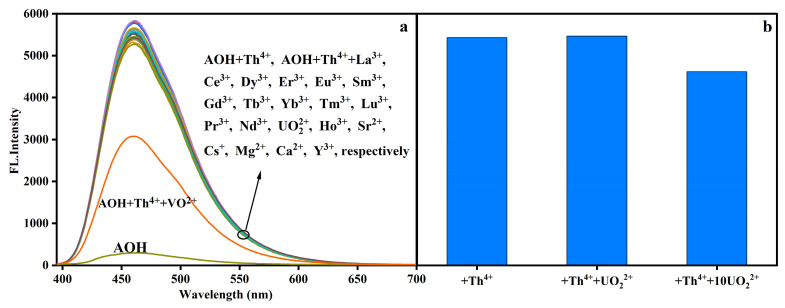
Fluorescence spectra of **AOH** (5 μM) at the coexistence of Th^4+^ and other metal ions in EtOH/H_2_O (1/1, *v*/*v*, pH = 2.0). (**a**) Th^4+^ (10 equiv., 50 μM) coexisted with other metal ions (10 equiv., 50 μM). (**b**) Effect of coexisted UO_2_^2+^ at different concentrations (10 equiv., 50 μM, and 100 equiv., 500 μM) with Th^4+^ (10 equiv., 50 μM).

Real Th^4+^-containing wastes (nuclear waste) contain a large amount of actinide and lanthanide ions in highly acidic environments [38,39]. The detection of Th^4+^ thus requires sensors exhibiting good anti-interference properties under acidic conditions. Here, the detection abilities of **AOH** toward Th^4+^ in the presence of competing metal ions were investigated (Figure 3). **AOH** shows excellent anti-interference ability toward Th^4+^ in the coexistence of tested metal ions except VO^2+^ (10 equiv.). Even though **AOH** presents distinguishable emission when VO^2+^ (1 equiv.) coexisted with Th^4+^ (10 equiv.) (Figure S6). Interestingly, **AOH** has an extreme resistance ability to f-block elements, especially to uranyl ions. It shows nearly no interference when 10 folds of UO_2_^2+^ and Th^4+^ are mixed with 1 equivalent of **AOH**. In addition, there was no substantial decline of the fluorescence intensity even when the concentration of UO_2_^2+^ enlarged to 100 folds over that of **AOH** (Figure 3b), superior to reported probes [40,41,42]. That will provide the possibility for further separation of the two nuclides using **AOH**.

### 2.3. Limit of Detection (LOD) on Th^4+^ Response

**Figure 4:**
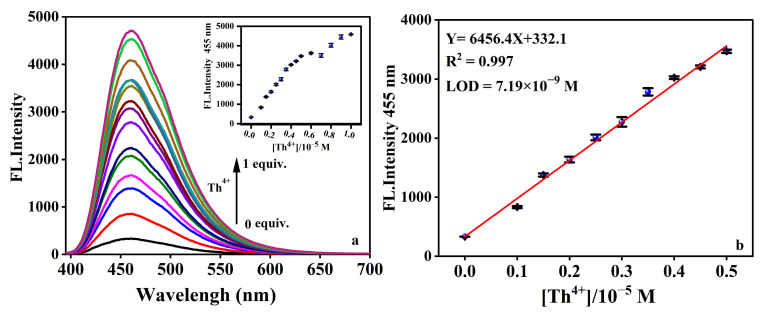
(**a**) Fluorescence spectra of **AOH** (10 μM) with gradual addition of Th^4+^ (0–10 μM) in EtOH/H_2_O (1/1, *v*/*v*, pH = 2.0). Inset is the corresponding fluorescence intensity at 455 nm with the concentration change of Th^4+^. (**b**) Calibration curve of the titration experiments.

The LOD for Th^4+^ was determined by titration experiments. An increase of the fluorescence emission with a maximum at 455 nm was observed during the gradual addition of Th^4+^. A linear relationship with the amount of added thorium was seen in the range of 0–5 μM (Figure 4).

**AOH** has a LOD of 7.19 nM for Th^4+^ in EtOH/H_2_O (1/1, *v*/*v*, pH = 2.0), calculated by the formula 3α/k (α is the standard deviation of **AOH** fluorescent intensity at 455 nm, k is the slope value in Figure 4b) [19,41]. Its limit of quantitation (LOQ, calculated by the formula 10α/k) for Th^4+^ is 24.1 nm. Both the LOD and LOQ are much lower than the World Health Organization (WHO) limits for thorium in drinking water (246 μg/L; 1.06 μM) [43]. Considering the tests were conducted in a benign aqueous solution, it is an improvement for practical Th^4+^ detection [16,44,45].

Meanwhile, the LOD of **AOH** to Th^4+^ with better water tolerance is lower than that of a similar sensor, N′-(2,4-dihydroxybenzylidene)-4-fluorophenyl hydrazide (**AF**, where the hydroxyl in **AOH** changed into fluorine in **AF**), we reported previously [46]. Under the same test condition for Th^4+^ response, **AOH** is superior to **AF** with over 2 folds of fluorescence enhancement (Figure S7). That is probably caused by the hydroxyl group in the structure of **AOH** which may not only take part in Th^4+^ detection but also may improve its solubility in water.

**Figure 5:**
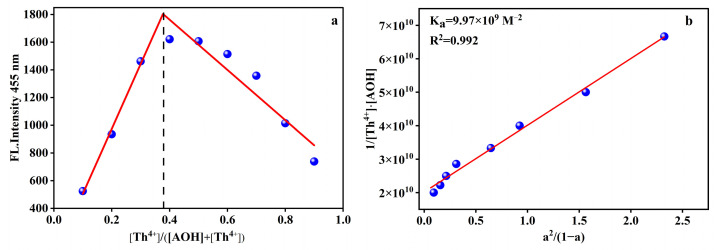
(**a**) The Job’s plot of **AOH**–Th^4+^. (**b**) Binding constant (*K*_a_) determination of **AOH** toward Th^4+^.

The Job’s plot (Figure 5a) shows a 2:1 binding ratio of **AOH**: Th^4+^, in accordance with the HRMS result (ESI^+^, [2**AOH** + Th^4+^ + NO_3_^−^ − O − H^+^]: experimental m/z 819.1680, calculated m/z 819.1789) (Figure S8). Based on this ratio, a binding constant value of 9.97 × 10^9^ M^−2^ was determined [41,47].

### 2.4. Selectivity on VO^2+^ Response

**Figure 6:**
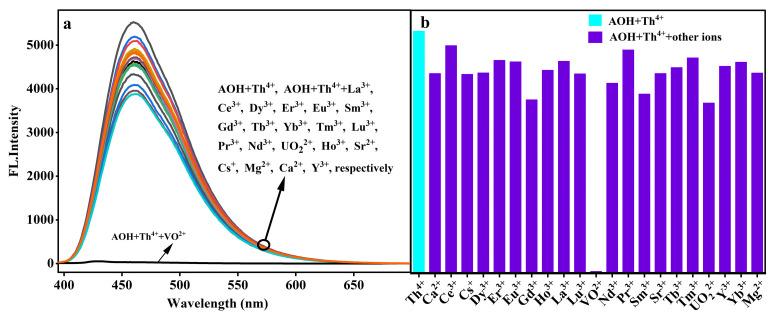
(**a**) Fluorescence spectra of **AOH** (5 μM) at the coexistence of Th^4+^ (10 equiv., 50 μM) and other metal ions (100 equiv., 500 μM) in EtOH/H_2_O (1/1, *v*/*v*, pH = 2.0). (**b**) The corresponding fluorescence response at 455 nm.

Compared to other metal ions in the above anti-interference tests shown in Figure 3, only VO^2+^ caused a decrease in fluorescence when it coexisted with Th^4+^ and **AOH**, and even the week fluorescence of **AOH** itself was quenched by VO^2+^ alone (Figure S9). Based on these results, the selective response of **AOH** to VO^2+^ with the help of Th^4+^ was tested among 21 metal ions including f-block ones (Figure 6). The results showed that the fluorescence was quenched especially when VO^2+^ coexisted with **AOH** and Th^4+^, whereas other metal ions cannot quench the luminescence of the **AOH**-Th^4+^ system. This indicates that **AOH**-Th^4+^ can be used as a selective sensor for the targeted detection of VO^2+^.

### 2.5. Interfering Ions Effect on VO^2+^ Response

**Figure 7:**
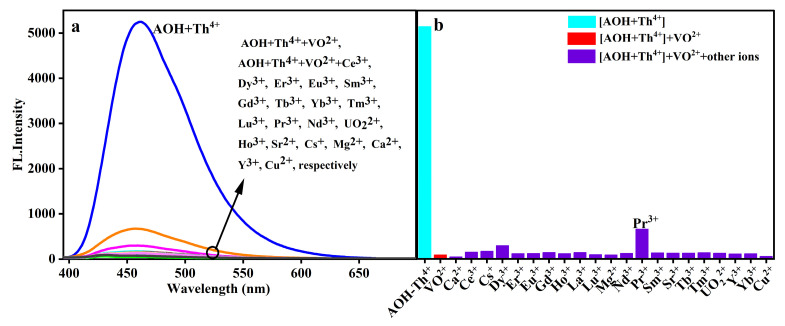
(**a**) Fluorescence spectra of **AOH** (5 μM)-Th^4+^ (10 equiv., 50 μM) in EtOH/H_2_O (1/1, *v*/*v*, pH = 2.0) coexists with VO^2+^ (50 equiv., 250 μM) and other metal ions (50 equiv., 250 μM). (**b**) Corresponding fluorescence intensities at 455 nm.

The **AOH**-Th**^4+^** system showed specific fluorescence “turn-off” towards VO^2+^. In anti-jamming effect tests, when other metal ions were mixed with VO^2+^ respectively, the luminescence was still not recovered (Figure 7). It is worth mentioning that Cu^2+^ [48,49], which has been reported to interfere with VO^2+^ detection, shows no obvious interference effect on the detection of VO^2+^ by **AOH**-Th^4+^. The system also shows different response to VO^2+^ and Fe^3+^ [50] (Figure S10), which demonstrates that **AOH**-Th^4+^ can be used as a new type of VO^2+^ fluorescence sensor with good anti-interference ability.

### 2.6. LOD of VO^2+^

**Figure 8:**
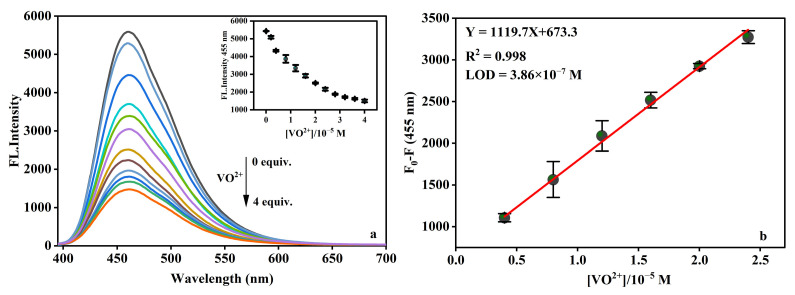
(**a**) Fluorescence spectra **AOH** (10 μM) -Th^4+^ (2 equiv., 20 μM) with gradual addition of VO^2+^ into EtOH/H_2_O (1/1, *v*/*v*, pH = 2.0). Inset is a scatter plot of the corresponding fluorescence intensity at 455 nm with the concentration increase of VO^2+^. (**b**) Linear relationship between VO^2+^ concentration and fluorescence intensity.

Since VOSO_4_ is the only salt of VO^2+^ we could obtain from chemical commercial companies, to prevent the effect of SO_4_^2−^, 10 folds of BaCl_2_ which has no effect on the fluorescence intensity of **AOH**-Th^4+^ (Figure S11), was added in the process during the titration tests to determine the LOD of the **AOH**-Th^4+^ system to VO^2+^. As shown in Figure 8a, the fluorescence intensity decreases gradually as the concentration of VO^2+^ increases from 0 to 4 equiv. of **AOH**. The change of fluorescence intensity at 455 nm is linearly related to the VO^2+^ concentration in the range of 4 to 24 µM (Figure 8b). The calculated detection limit of **AOH**-Th^4+^ to VO^2+^ is 0.386 µM (31 µg/L), which is much lower than the reference human risk dose of 350 µg/d for vanadium for an adult with a weight of 50 kg (7 µg/kg/d, USEPA. 2021) [51].

The Job’s plot shows a 1:1 binding ratio of **AOH**-Th^4+^: VO^2+^ (Figure S12a). Based on that, the binding constant is calculated to be 4.54 × 10^4^ M^−1^ (Figure S12b) [19].

### 2.7. Recognition Mechanism of AOH on Th^4+^ and VO^2+^ Response

**Figure 9:**
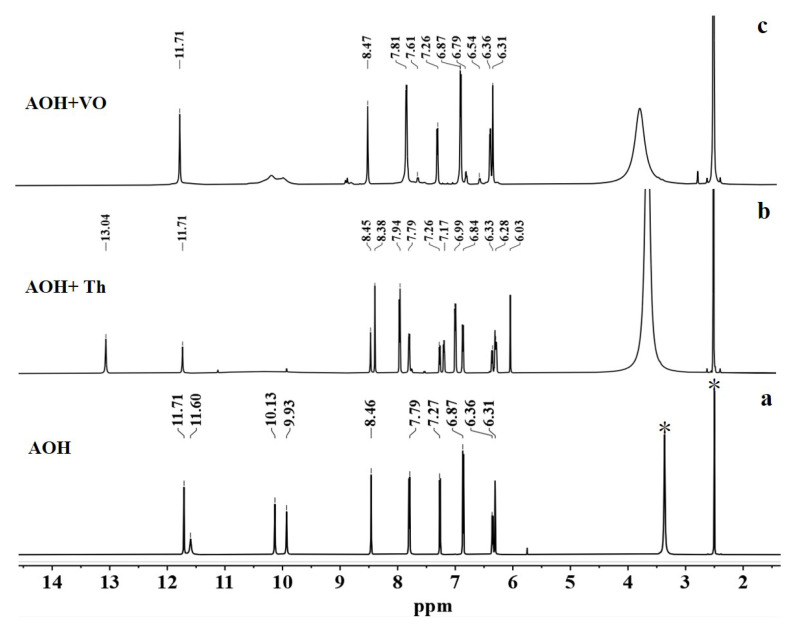
^1^H NMR spectra of **AOH** before (**a**) and after (**b**) Th^4+^ and (**c**) VO^2+^ addition. The asterisks stand for the solvent signals.

In order to understand the detection mechanisms, the ^1^H NMR spectra of **AOH** before and after Th^4+^ and VO^2+^ blending were compared (Figure 9). The results show that there was no change in the proton hydrogen signal (δ 11.71 ppm) of -NH-, either on addition of Th^4+^ or VO^2+^, indicating that the nitrogen of the amide group in **AOH** was not involved in the detection. The addition of Th^4+^ caused the hydrogen signals (δ 11.60, 10.13, 9.93 ppm) of -OH groups in **AOH** to move to lower field or damped significantly (Figure 9b). Meanwhile, the hydrogen of -CH=N- shifted from 8.45 ppm to 8.38 ppm with different extent of shifts of the hydrogens in the aromatic rings of the sensor, indicating their involvements in the binding with Th^4+^. The FT-IR spectra changes of C=O (1602 cm^−1^ to 1614 cm^−1^) and OH (1359 cm^−1^ to 1384 cm^−1^) demonstrate that the oxygens are involved in the reaction of **AOH** and Th^4+^ (Figure S13). The broad or disappearing signals of all the hydrogens in -OH groups of **AOH** after VO^2+^ addition (Figure 9c) illustrates the oxygen participation in the complexing reaction. The minor shifts of the residual hydrogens in **AOH** demonstrate the different combination modes of VO^2+^ to Th^4+^ with **AOH**.

**Figure 10:**
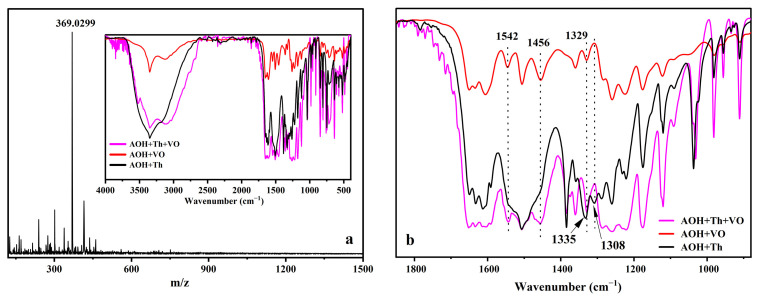
(**a**) HRMS spectrum of **AOH** mixed with Th^4+^ and VO^2+^. Insets are the full FT-IR spectra of **AOH** before and after mixing with Th^4+^ and VO^2+^ respectively. (**b**) The enlarged FT-IR spectra of **AOH** before and after it was mixed with Th^4+^ and VO^2+^ at 1800–850 cm^−1^.

When **AOH** was mixed with Th^4+^ and VO^2+^, the corresponding HRMS spectrum shows the main m/z at 369.0299 (Figure 10a), attributed to the signal of **AOH**-VO^2+^ with a 1:1 ratio ([**AOH** + VO^2+^ + CH_3_O^−^ − H^+^], calculated: *m*/*z* 369.0291), indicating that Th^4+^ is not involved in the binding and that the nuclide ion coordinated to **AOH** was replaced by VO^2+^. This speculation was verified by the FT-IR results when **AOH** was mixed with Th^4+^ or VO^2+^ or their mixture (Figure 10b). The signals at 1542 cm^−1^ and 1456 cm^−1^ appeared clearly in the mixed sample of **AOH** with Th^4+^ and VO^2+^, which correspond to the characteristic absorption peaks in **AOH**-VO^2+^. In addition, the peak at 1335 cm^−1^ in **AOH**-Th^4+^ was shifted to 1329 cm^−1^ of **AOH**-VO^2+^, and the signal at 1308 cm^−1^ corresponding to the absorption band of **AOH**-Th^4+^ disappeared when VO^2+^ was added in the sample. These changes partly explain the reason for fluorescence quenching of **AOH**-Th^4+^ when VO^2+^ coexisted with it.

**Figure 11:**
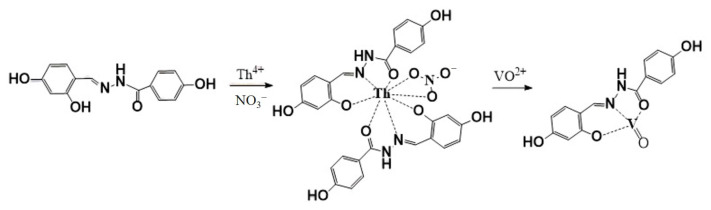
Proposed complexing mechanism of **AOH** with Th^4+^ and VO^2+^.

Based on the above test analyses, the sensing mechanism of **AOH** to Th^4+^ and VO^2+^ is proposed as shown in Figure 11.

**Figure 12:**
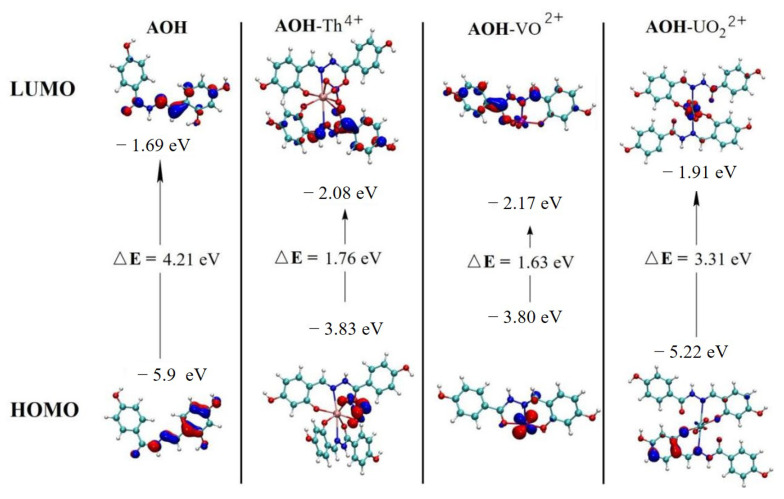
The frontier molecular orbitals of **AOH** and **AOH**-Th^4+^, **AOH**-VO^2+^ and **AOH**-UO_2_^2+^ complexes.

To further understand the response mechanism, the optimized structure of **AOH** and **AOH**-metal ion complexes were calculated by density functional theory (DFT) at B3LYP/6-311G (d, p) level using Gaussian 09 software. In this process, the metal atoms used are described using Stuttgart small-core pseudopotentials and basis sets [52]. Subsequently, based on the wave function information, Multiwfn 3.8(dev) software [53,54] was used to calculate the HOMO and LUMO orbitals. Their frontier molecular orbitals are shown in Figure 12.

In the free ligand, the HOMO is located at the imine groups and dihydroxybenzene ring respectively, while the LUMO is distributed between the imine groups and dihydroxybenzene ring, indicating the intramolecular charge transfer (ICT) effect in **AOH**. After its combination with Th^4+^, the LUMO is moved to the hydrazone bond with the benzamide ring that belongs to one of the two **AOH**s, suggesting the block of ICT process and the enhancement of fluorescence [55]. The calculated HOMO-LUMO energy gap in **AOH-**Th^4+^ (1.76 eV) is much lower than that in free ligand (4.21 eV), indicating that **AOH** is reactive to Th^4+^. On the other hand, although the energy gap in **AOH**-UO_2_^2+^ (3.31 eV) is a bit lower than that of the free ligand, it is still much higher than 1.76 eV in **AOH**-Th^4+^, demonstrating reaction precedence of **AOH** with Th^4+^ over UO_2_^2+^. That explains the excellent anti-uranyl ability of **AOH** in Th^4+^ detection when it coexists with UO_2_^2+^. The energy gap in **AOH**-VO^2+^ (1.63 eV) is even a bit lower than that in **AOH**-Th^4+^, indicating that **AOH** will react with VO^2+^ if its amount is over than that of Th^4+^, which is consistent with the experimental results. The HOMO of **AOH**-Th^4+^ is located on the nitrate whereas it is located on VO^2+^ of **AOH**-VO^2+^, which in part explains the experimentally observed phenomena of fluorescence “turn-on” in the former and “turn-off” in the latter.

### 2.8. Detection Comparison to Reported Sensors on Th^4+^ or VO^2+^

As we have not found other probes that work on Th^4+^ and VO^2+^ detection, here, we list in Table 1 some related chemical sensors reported on Th^4+^ or VO^2+^ for comparison. Considering various factors such as testing media, binding constant, detection limit, and anti-interference abilities, **AOH**, with its easy accessibility, can be a suitable candidate for Th^4+^ and VO^2+^ analysis.

### 2.9. Th^4+^ and VO^2+^ Determination in Real Water

The detection applications of **AOH** towards Th^4+^ and VO^2+^ in real water samples were tested and the results are listed in Table 2 and Table 3. Either in tap water or in pool water, Th^4+^ and VO^2+^ had good recovery with less than 1.5% mean relative standard deviation (RSD), indicating good real water application prospects of **AOH** in Th^4+^ and VO^2+^ detection. Anion influence tests showed negligible effect on Th^4+^ and VO^2+^ detection (Figure S14), supporting its application ability in real conditions.

## 3. Materials and Methods

### 3.1. Materials and Reagents

All analytical grade reagents were purchased from the Aladdin-Reagent or Tansoole company (Shanghai, China) and used directly without further purification. The corresponding metal ion stock solutions were prepared by dissolving LaCl_3_, CeCl_3_, Pr(NO_3_)_3_, Nd(NO_3_)_3_, LuCl_3_, TbCl_3_, EuCl_3_, Er(NO_3_)_3_, Yb(NO_3_)_3_, Tm(NO_3_)_3_, Gd(NO_3_)_3_, Sm(NO_3_)_3_, Dy(NO_3_)_3_, Ho(NO_3_)_3_, UO_2_(NO_3_)_2_, VOSO_4_, CuCl_2_, BaCl_2,_ and FeCl_3_, respectively in ultrapure water. Thorium standard solution was purchased from Weiye Measurement Co. (Xinyang city, Henan, China) at a concentration of 1000 μg/mL (5% of HNO_3_ in volume). NaF, NaCl, KNO_3_, K_2_SO_4_, and KBr were used for anions effect tests. The desired pH solutions were obtained by adding dilute HNO_3_ or NaOH to pure water. The water was deionized to a specific resistivity of 18.2 MΩ cm using the ULUPURE Water Purification System.

**CAUTION:** Uranyl and thorium ions have a certain level of radioactivity. Appropriate protective equipment should be worn during operation, such as laboratory work clothes, gloves, and goggles, to reduce the risk of direct contact and inhalation.

### 3.2. Synthesis of AOH

**Scheme 1:**
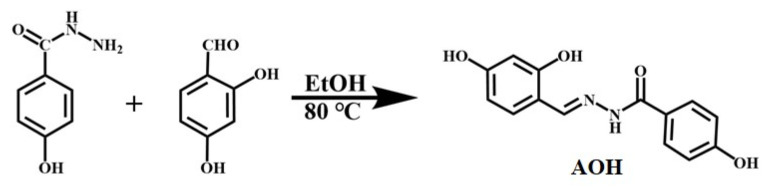
The synthesis route of **AOH**.

**AOH** (Scheme 1) was synthesized based on modified processes of our former work **[58].** Typically, 1 mmol (0.156 g) of 4-Hydroxybenzhydrazide was dissolved in 15 mL of ethanol. Then 1 mmol (0.140 g) of 2,4-dihydroxybenzaldehyde in 10 mL of ethanol was added into the above solution. The reaction mixture was stirred at 80 °C for 6 h and the reaction process was monitored by TLC. After the reaction, a large amount of white solid precipitated from the solution. It was collected by filtration and purified by re-crystallization from 40 mL of hot ethanol to obtain the target product: white solid, 0.202 g, yield: 74%. The synthesis route is shown in Scheme 1. ^1^H NMR, UV-vis, HRMS, and IR spectra (Figures S1 and S2) verified its excellent purity and its structure [34].

^1^H NMR of **AOH** (600 MHz, DMSO-*d_6_*, Figure S1): δ 11.71 (s, 1H, -NH), δ 11.60 (s, 1H, -OH), δ 10.13 (s, 1H, -OH), δ 9.93 (s, 1H, -OH), δ 8.46 (s, 1H, -CH=N), δ 7.81 (d, 2H, ArH), δ 7.27 (d, 1H, ArH), δ 6.86 (d, 2H, ArH), δ 6.35 (q, 1H, ArH), δ 6.31 (d, 1H, ArH).

IR of **AOH** (ν, cm^−1^, Figure S2b): 3345 (-OH), 3123 (N-H), 1602 (C=O), 1542 (C=N).

### 3.3. Apparatus

^1^H NMR measurements were performed on a Bruker Avance III 600 MHz system (Billerica, MA, US), using DMSO-*d*_6_ as the solvent with TMS as the internal standard. The pH adjustments were performed by a Thermo Fisher Scientific (model XL200, Shanghai, China) digital pH meter equipped with a combination glass electrode. Fluorescence spectra were recorded on a F-4600 spectrophotometer (Tokyo, Japan) with a quartz cuvette (path length = 1 cm) with an excitation of 374 nm. The excitation and emission bandwidths were both 5.0 nm unless otherwise noted. FT-IR spectra of the samples (KBr disk) were recorded on a Bruker (Billerica, MA, USA) FT-IR spectrometer in the range of 4000~400 cm^−1^. ESI-HRMS spectra were recorded on an Agilent 6224 (Waldbronn, Germany) TOF MS spectrometer or Waters (Milford, MA, USA) HClass MS spectrometer.

### 3.4. Solution Preparation and Testing Process

**AOH** was dissolved in ethanol to obtain a stock probe solution (1 mM). The corresponding metal salts were dissolved in pure water to obtain the corresponding metal ion stock solutions (10 mM). The standard solution of thorium nitrate was diluted with pure water to obtain a stock solution of Th^4+^ (4 mM), and a stock solution of UO_2_^2+^ (10 mM) was prepared by dissolving uranyl nitrate hexahydrate in pure water. All of the stock solutions were used within one month.

Analysis procedures: The selectivity and anti-interference abilities to metal ions were tested in the media of EtOH/H_2_O (1/1, *v*/*v*, pH = 2.0). The probe stock solution (1 mM) was mixed with metal ion salt (10 equiv.) solution and diluted with ethanol and water to 5 μM. In anti-interference tests, Th^4+^ ions (10 equiv.) and interfering metal ions (10 equiv.) were added to **AOH** (1 mM) and diluted to 5 μM of **AOH** (EtOH/H_2_O, 1/1, *v*/*v*, pH = 2.0). In evaluating the anti-interference performance of VO^2+^, the probe (5 μM), Th^4+^ ions (10 equiv.), interfering ions (50 equiv.), and VO^2+^ (50 equiv.) were mixed and then diluted to the target concentrations for tests. The samples were placed at room temperature for 30 min before fluorescence testing.

In real water tests, the lake water was taken from the lake of Southwest University of Science and Technology, Mianyang, China. The lake water sample was filtered by a 0.45 μm membrane before use. Tap water in the lab was used directly. The water pH values were adjusted to 2.0 with HCl and mixed with ethanol in an equal volume.

## 4. Conclusions

Acylhydrazone, **AOH**, a small drug molecule, was first developed for sequential Th^4+^ and VO^2+^ monitoring in an acidic aqueous solution (EtOH/H_2_O, 1:1, *v*/*v*, pH = 2.0) without changing the medium conditions. Its specific luminescence enhancement to Th^4+^ and subsequent fluorescence quenching to VO^2+^ (more notable at the coexistence of Th^4+^), demonstrate its value as a chemosensor towards the two ions responses. In the presence of disturbing lanthanide ions and the most competing uranyl ions, **AOH** has excellent anti-interference abilities during Th^4+^ and VO^2+^ detection. The presented results provide the possibility of the nuclide ions detection and future separation in a simple way.

## Figures and Tables

**Table 1:** Fluorescent recognition comparison of **AOH** towards Th^4+^ or VO^2+^ with some reported sensors in aqueous solutions.

Sensor (Ref.)	Detecting Object	Media (*v*/*v*)	K_a_	LOD	Interfering Ions
[19]	Th^4+^	MeOH/H_2_O (7:3)	2.03 × 10^4^ M^−1^	20.0 nM	UO_2_^2+^, Cu^2+^
[41]	Th^4+^	MeOH/H_2_O (7:3)	2.46 × 10^4^ M^−1^	34.4 nM	UO_2_^2+^
[56]	Th^4+^	CH_3_CN/H_2_O (9:1)	5.67 × 10^3^ M^−1^	2.10 nM	Fe^3+^
[57]	Th^4+^	MeOH/H_2_O (1:1)	NR	0.60 μM	Cu^2+^, Fe^3+^, UO_2_^2+^, Lu^3+^
[46]	Th^4+^	EtOH/H_2_O (7:3, pH 2.0)	6.64 × 10^9^ M^−2^	29.2 nM	NO
[48]	VO^2+^	DMSO-Tris-HCl (7:3, pH 7.4)	NR	3.65 nM	Cu^2+^
[50]	VO^2+^	H_2_O	4.40 × 10^3^ M^−1^	0.34 μM	Fe^3+^
This work	Th^4+^, VO^2+^	EtOH/H_2_O (1:1, pH 2.0)	9.97 × 10^9^ M^−2^ 4.54 × 10^4^ M^−1^	7.19 nM 0.386 μM	NO NO

NR: not reported; NO: No interference.

**Table 2:** Determination of Th^4+^ by **AOH** (20 μM) in different water samples.

Sample	Th^4+^ Added (μM)	Th^4+^ Found (μM)	Recovery (%)	RSD (%)
Tap water	25.0	25.0	100.0	0.9
	25.0	25.3	101.2	
	25.0	24.8	99.3	
Pool water	25.0	24.9	99.5	0.9
	25.0	25.3	101.3	
	25.0	25.1	100.3	

**Table 3:** Determination of VO^2+^ by **AOH** (20 μM) -Th^4+^ (20 μM) in different water samples.

Sample	Th^4+^ Added (μM)	Th^4+^ Found (μM)	Recovery (%)	RSD (%)
Tap water	25.0	24.9	99.8	0.6
	25.0	25.1	100.3	
	25.0	25.3	101.0	
Pool water	25.0	25.1	100.4	1.2
	25.0	25.7	102.7	
	25.0	25.3	101.0	

## Data Availability

Experimental data are available from the authors upon reasonable request.

## Supplementary Materials

The following supporting information can be downloaded at: https://www.mdpi.com/article/10.3390/ijms26073231/s1.

## Abbreviations

The following abbreviations are used in this manuscript:

AOH	Acylhydrazone, N’-(2,4-dihydroxybenzylidene)-4-hydroxylphenylhydrazide
ICT	Intramolecular charge transfer
LOD	Limit of detection
