# Supplementary material for: An Acylhydrazone Fluorescent Sensor: Bifunctional Detection of Thorium (IV) and Vanadyl Ions over Uranyl and Lanthanide Ions
Source: Int J Mol Sci. 2025 Mar 31;26(7):3231. doi: 10.3390/ijms26073231 (PMC11989325; doi:10.3390/ijms26073231)
Supplement: Supplementary file 1 [file ijms-26-03231-s001.zip › ijms-3551051-supplementary.pdf]

Supporting information

Table of Contents

Figure S1. ^{1}H NMR spectrum of **AOH**.

Figure S2. HRMS, UV-vis and IR spectra of **AOH**.

Figure S3. pH effect on **AOH** response to Th^{4+}.

Figure S4. Stability test of **AOH** in acidic condition.

Figure S5. Selectivity of **AOH** to Th^{4+} over other common metal ions.

Figure S6. The coexistence effect of VO^{2+} and Th^{4+} at different molar ratio.

Figure S7. Response comparison of **AOH** with its analogue in Th^{4+} response.

Figure S8. HRMS spectrum of **AOH**- Th^{4+}.

Figure S9. Fluorescence spectra of **AOH** alone and after the addition VO^{2+}.

Figure S10. Coexisting ion effect (VO^{2+}, Cu^{2+}, and Fe^{3+}) on the fluorescence spectra of **AOH-**Th^{4+}.

Figure S11. Coexisting Ba^{2+} influence on the fluorescence spectra of **AOH**-Th^{4+}.

Figure S12. The job's plot of **AOH-**$\mathbf{Th^{4+}}$ to VO^{2+} and binding constant determination.

Figure S13. FT-IR spectrum comparison of **AOH** before and after Th^{4+} addition.

Figure S14. Anions effect on Th^{4+} and VO^{2+} detection.

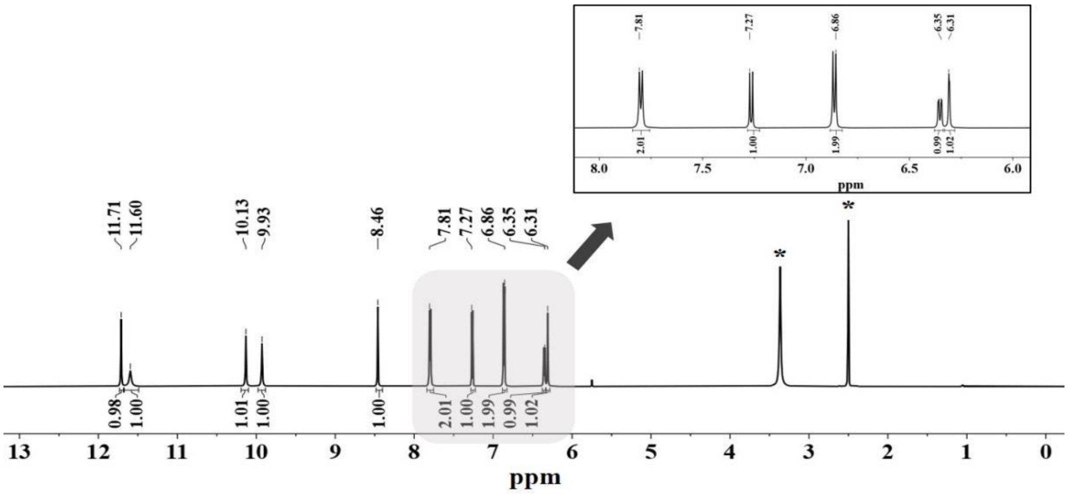

Fig. S1. ^{1}H NMR spectrum of **AOH** in DMSO-d_6 (the asterisks show the solvent peaks).

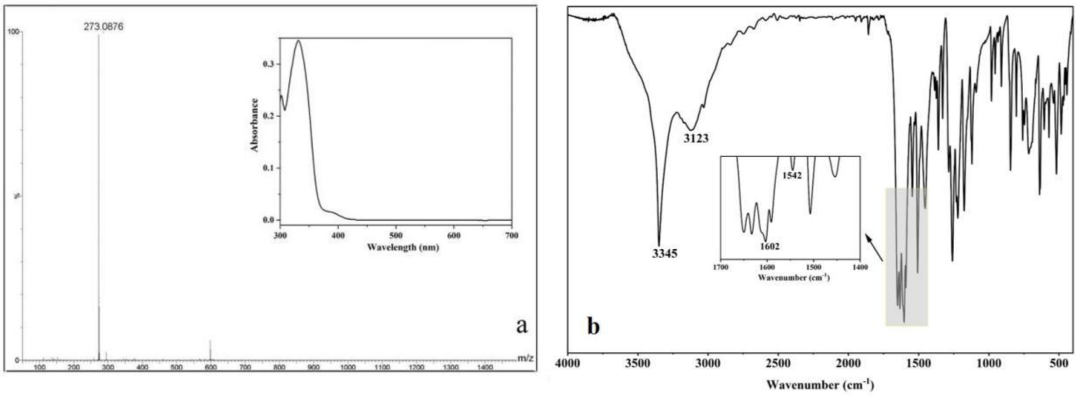

Fig. S2. (a) ESI-HRMS spectrum (inset is the UV-vis spectrum in ethanol) and (b) FTIR spectrum of **AOH**

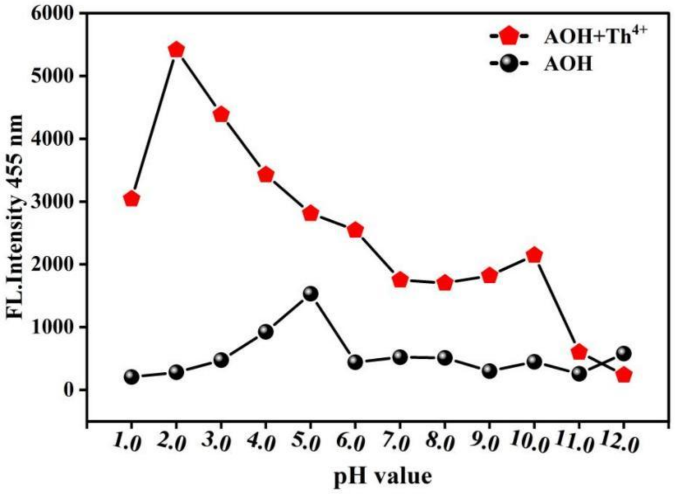

Fig. S3. The pH effects on fluorescence intensity of **AOH** with Th^{4+} respectively in EtOH/H_2O (1/1, v/v).

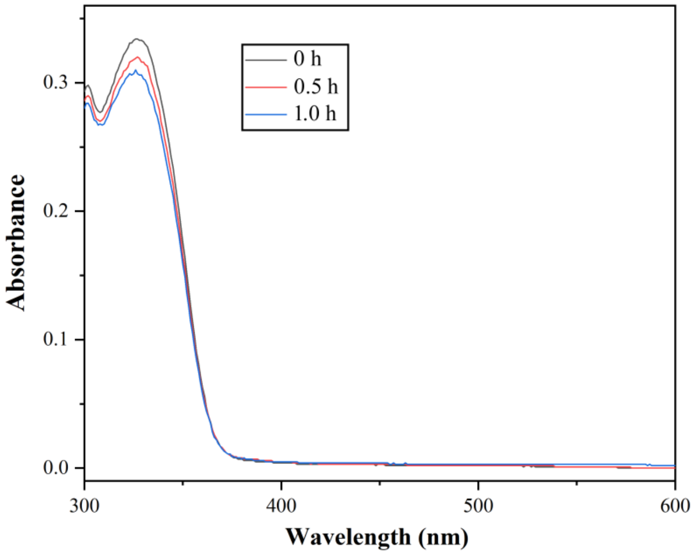

Fig. S4. The UV-vis spectra of **AOH** at different time in EtOH/H_2O (1/1, v/v, pH = 2.0).

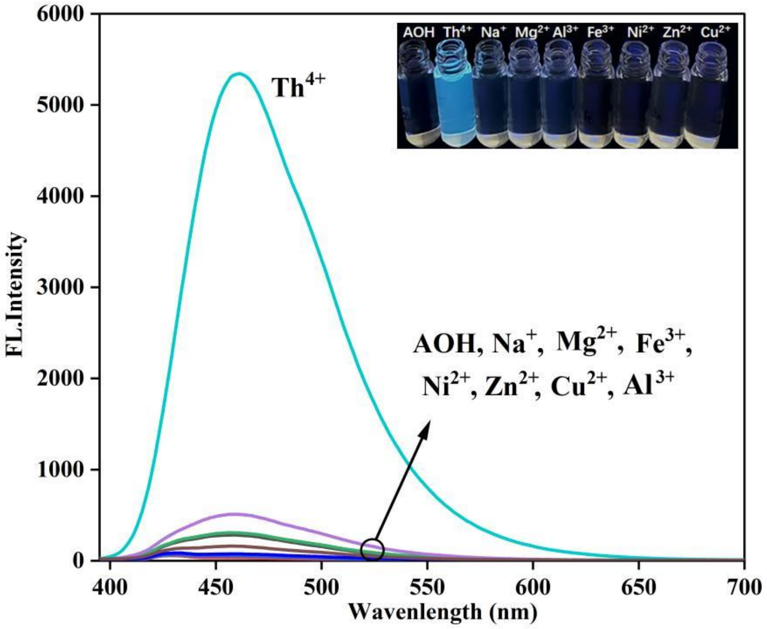

Fig. S5. Fluorescence selectivity of **AOH** (5 μM) to Th^{4+} (50 μM) among common metal ions (50 μM) in EtOH/H_2O (1/1, v/v, pH = 2.0). Inset is the corresponding image under 365 nm UV light.

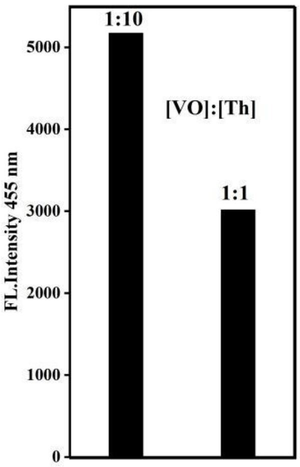

Fig. S6. The fluorescence intensity of VO^{2+} (2 μM and 20 μM, respectively) and Th^{4+} (20 μM) coexisting with **AOH** (10 μM) at different molar ratios.

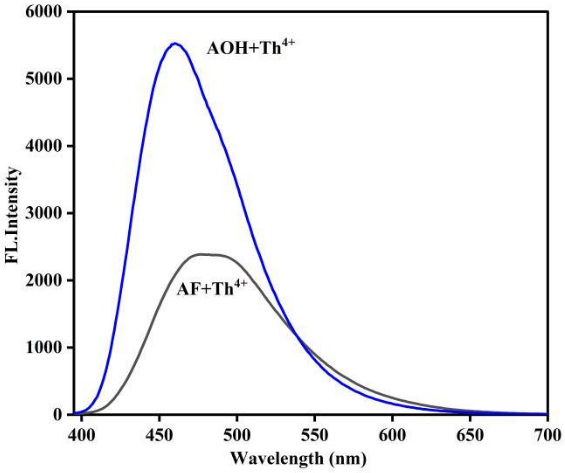

Fig. S7. Fluorescence spectrum comparison of **AOH** (5 μM) and our previous sesor **AF** (5 μM) in the existence of Th^{4+} (50 μM) at the same conditions in $EtOH/H_2O$ (1/1, v/v, pH = 2.0).

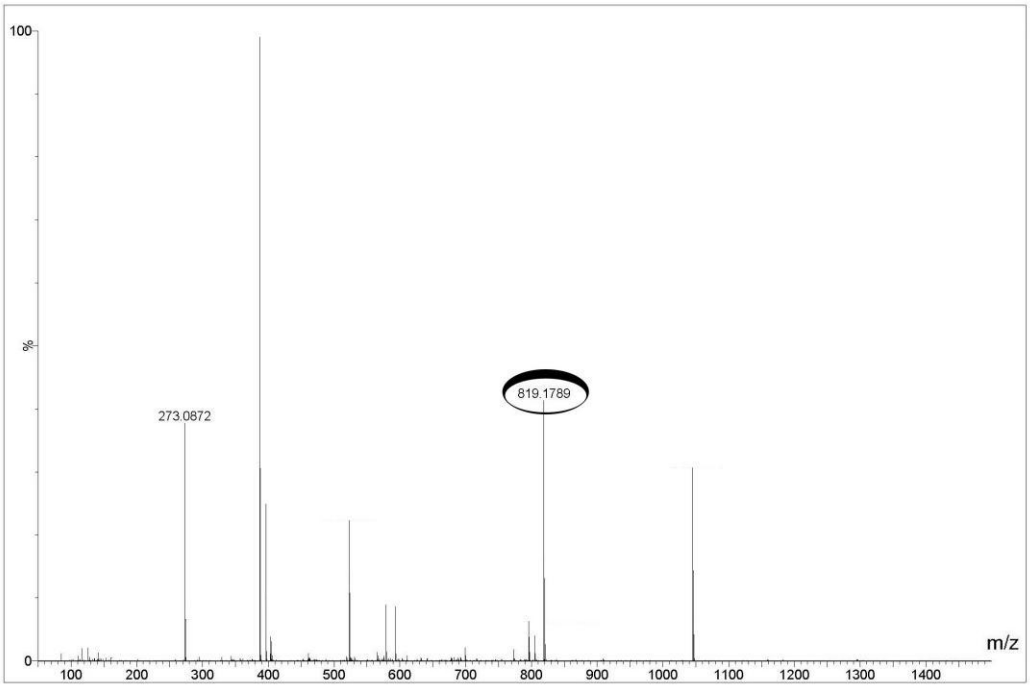

Fig. S8. HRMS spectrum of **AOH** mixed with Th^{4+}

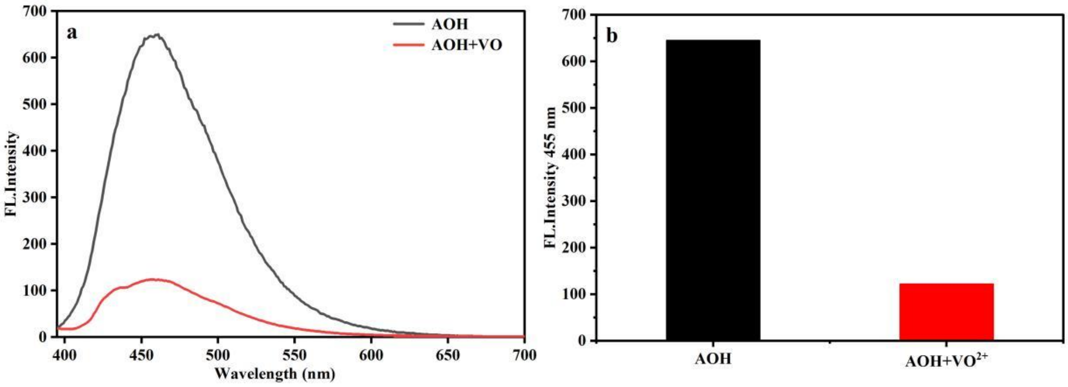

Fig. S9. (a) Fluorescence spectra of **AOH** (5 μM) alone and after the addition VO^{2+} (50 μM) in EtOH/H_2O (1/1, v/v, pH = 2.0). (b)The corresponding fluorescence intensity histogram at 455 nm

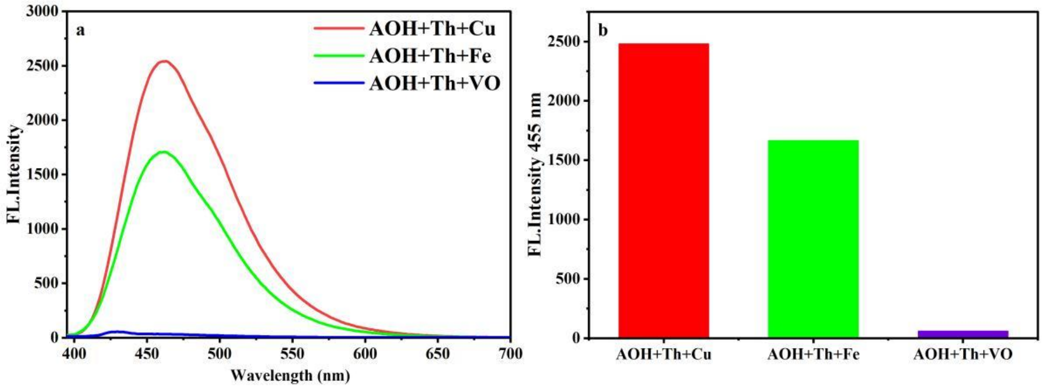

Fig.S10. (a) Fluorescence spectra of **AOH** (5 μM)-Th^{4+} (50 μM) coexisted with VO^{2+} (500 μM), Cu^{2+} (500 μM) and Fe^{3+} (500 μM) respectively in EtOH/H_2O (1/1, v/v, pH = 2.0). (b) The corresponding fluorescence intensity histograms at 455 nm

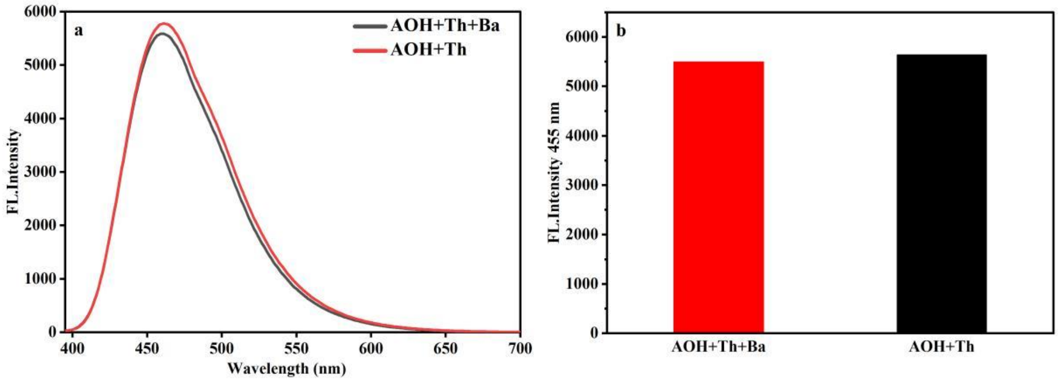

Fig. S11. (a) Fluorescence spectra of AOH (10 μM)-Th^{4+} (2 equiv., 20 μM) before and after adding $BaCl_2$ (10 equiv., 100 μM). (b) The corresponding fluorescence intensity histograms at 455 nm.

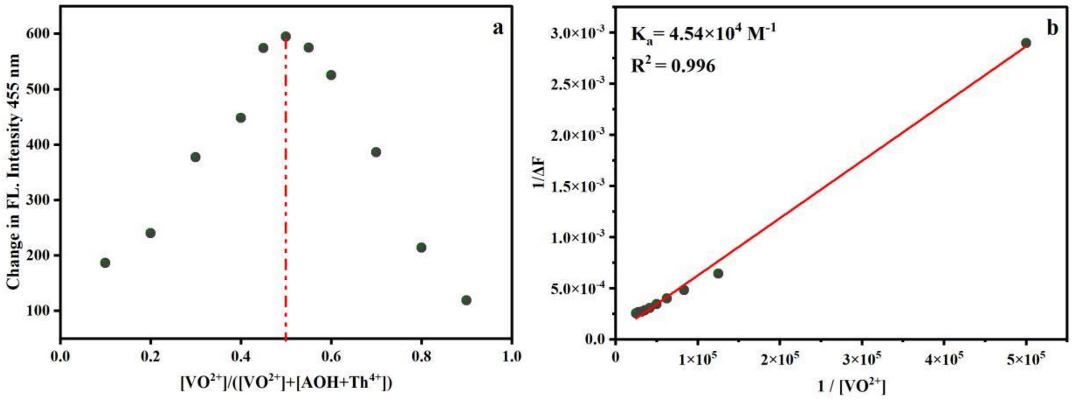

Fig. S12. (a) The job's plot of **AOH-Th^{4+}** to VO^{2+}. (b) Binding constant (K_a) determination

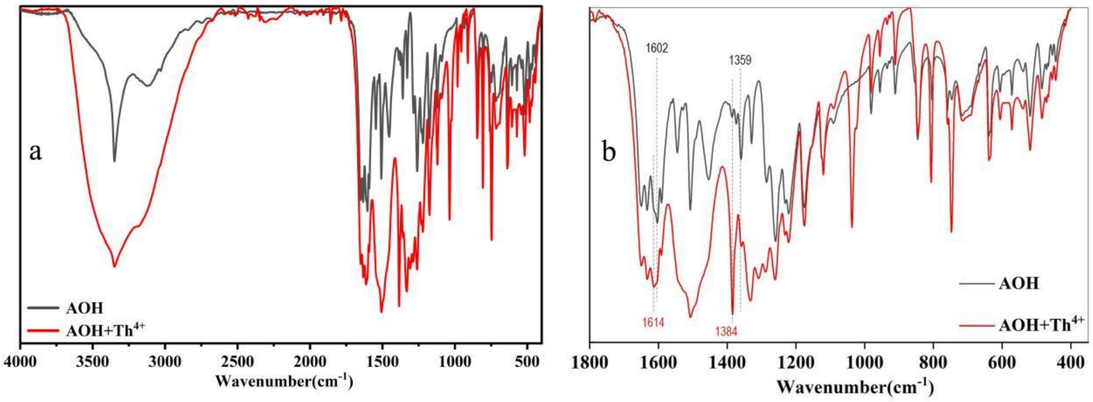

Fig. S13. FT-IR spectra of **AOH** before (black line) and after (red line) Th^{4+} addition. (a) The full spectrum and (b) the enlarged spectrum at 1800 - 400 cm^{-1}.

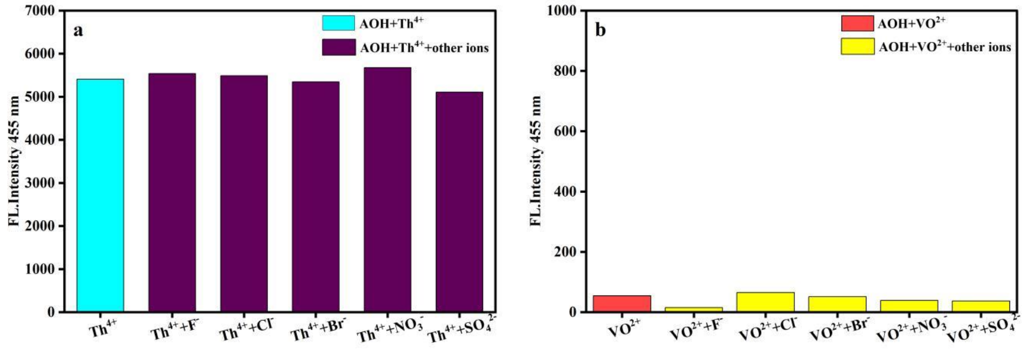

Fig. S14. Anions effect on the fluorescence intensity (455 nm) for Th^{4+} and VO^{2+} detection, with the coexistence of tested anions (50 μM) in EtOH/H_2O (1/1, v/v, pH = 2.0). (a) **AOH** (5 μM) -Th^{4+} (50 μM). (b) **AOH** (5 μM) -VO^{2+} (50 μM).
